# A sex difference in mouse dopaminergic projections from the midbrain to basolateral amygdala

**DOI:** 10.1186/s13293-022-00486-4

**Published:** 2022-12-30

**Authors:** Matthew T. C. Manion, Erica R. Glasper, Kuan Hong Wang

**Affiliations:** 1 Unit on Neural Circuits and Adaptive Behaviors, National Institute of Mental Health, National Institutes of Health, Bethesda, MD 20892 USA; 2 Department of Psychology, University of Maryland, College Park, MD 20742 USA; 3 Program in Neuroscience and Cognitive Science, University of Maryland, College Park, MD 20742 USA; 4 Department of Neuroscience and Institute for Behavioral Medicine Research, The Ohio State University, Columbus, OH 43235 USA; 5 Department of Neuroscience, Del Monte Institute for Neuroscience, University of Rochester Medical Center, Rochester, NY 14642 USA

**Keywords:** Dopamine, Basolateral amygdala, Sex difference, Axon projections, Synaptic boutons

## Abstract

**Background:**

Dopaminergic circuits play important roles in the motivational control of behavior and dysfunction in dopaminergic circuits have been implicated in several psychiatric disorders, such as schizophrenia and depression. While these disorders exhibit different incidence rates in men and women, the potential sex differences in the underlying neural circuits are not well-understood. Previous anatomical tracing studies in mammalian species have revealed a prominent circuit projection connecting the dopaminergic midbrain ventral tegmental area (VTA) to the basolateral amygdala (BLA), which is involved in emotional processing and associative learning. However, whether there is any sex difference in this anatomical circuit remains unknown.

**Methods:**

To study the potential sex differences in the VTA-to-BLA dopaminergic circuit, we injected two viral vectors encoding fluorescent reporters of axons and synaptic boutons (AAV–FLEX–tdTomato and AAV–FLEX–SynaptophysinGFP, respectively) into the VTA of a mouse transgenic driver line (tyrosine hydroxylase promoter-driven Cre, or TH-Cre), which restricts the reporter expression to dopaminergic neurons. We then used confocal fluorescent microscopy to image the distribution and density of dopaminergic axons and synaptic boutons in serial sections of both male and female mouse brain.

**Results:**

We found that the overall labeling intensity of VTA-to-BLA dopaminergic projections is intermediate among forebrain dopaminergic pathways, significantly higher than the projections to the prefrontal cortex, but lower than the projections to the nucleus accumbens. Within the amygdala areas, dopaminergic axons are concentrated in BLA. Although the size of BLA and the density of dopaminergic axons within BLA are similar between male and female mice, the density of dopaminergic synaptic boutons in BLA is significantly higher in male brain than female brain.

**Conclusions:**

Our results demonstrate an anatomical sex difference in mouse dopaminergic innervations from the VTA to BLA. This finding may provide a structural foundation to study neural circuit mechanisms underlying sex differences in motivational and emotional behaviors and related psychiatric dysfunctions.

## Highlights

- Cell-type specific labeling of VTA-to-BLA dopaminergic axons and synaptic boutons in mice
- The intensity of dopaminergic innervation in BLA is intermediate between those in PFC and NAC
- Dopaminergic axon density in BLA and the size of BLA are similar between males and females
- The density of dopaminergic boutons in BLA is significantly higher in males than females

## Background

Sex differences in behaviors are widely observed across mammalian species and well-recognized in human psychiatric disorders [1–4]. For example, major depression and anxiety disorders occur more frequently in women [5–7], whereas men show an increased incidence of schizophrenia and attention-deficit hyperactivity disorder (ADHD) [8–10]. To understand the neurobiological foundations of sex differences in psychiatric illnesses and related behavior patterns, it is necessary to investigate sex differences in brain circuits implicated in psychiatric dysfunctions through in-depth anatomical examination in model organisms [11–14].

Historically, studies of anatomical sex differences in the brain initially focused on neural regions strongly associated with reproductive behaviors, such as the hypothalamus [13, 15]. Consistently reported in several rodent species, the sexually dimorphic nucleus of the medial preoptic area of the hypothalamus (SDN–MPOA) has a larger volume in males, whereas the anteroventral periventricular nucleus (AVPV) of the hypothalamus contains a greater number of dopaminergic neurons in females [16–19]. In addition, brain regions in the limbic–hypothalamic sensory pathways, such as the medial amygdala (MEA) and the bed nuclei of the stria terminalis (BNST), show prominent anatomical sex differences [20–24].

Sex differences in limbic brain regions outside of the immediate reproductive behavior circuits—such as the basolateral amygdala (BLA), prefrontal cortex (PFC), and nucleus accumbens (NAC)—have also been reported using anatomical, molecular, or physiological measures [25–29]. These brain regions are engaged in emotional responses, decision-making, and goal-directed actions, which are commonly impaired in psychiatric disorders [30–32]. Moreover, all three of these brain regions receive prominent projections from the midbrain ventral tegmental area (VTA), which release dopamine as a critical neuromodulator of motivated behaviors [33–36]. In addition, dopaminergic dysfunction has been well-documented in psychiatric disorders, such as schizophrenia, ADHD, and mood disorders [31, 37, 38].

Of the three forebrain regions targeted by dopaminergic projections, BLA has a unique clinical relevance due to its role in affective processing and the reported difference in incidence rate of depression and anxiety between men and women [7, 39–41]. BLA is considered to play key roles in valence-specific responses and associative learning of both rewarding and aversive stimuli [42–45]. Anatomical tracer and viral labeling studies in mice indicate that BLA receives major dopaminergic inputs from VTA [34, 46–48]. Although dopaminergic projections to BLA have received much less research attention compared to projections to NAC or PFC, recent studies have suggested that VTA-to-BLA dopaminergic projections provide gating of salient sensory cues and regulate anxiety-related behaviors [35, 49, 50]. Interestingly, a greater level of extracellular dopamine has been reported in BLA of male rats than female rats [27]. However, whether an anatomical foundation exists for this physiological difference is unknown. Although several studies have provided fine-scale anatomical analyses of forebrain dopaminergic projections [46, 51, 52], these analyses have not focused on sex differences. To date, whether there is an anatomical sex differences in VTA-to-BLA dopaminergic projections remains unknown.

In this study, we used a combination of cell type-specific transgenic driver mice and viral vectors encoding fluorescent reporters of axons and synaptic boutons to label dopaminergic projections from the VTA to forebrain target regions, and specifically probed the anatomy of VTA-to-BLA circuit for sex differences. We hypothesized that males and females would show different degrees of dopaminergic innervation in VTA projection target regions. We observed an anatomical difference in the number of dopaminergic synaptic boutons between males and females, with males showing a higher density of boutons in the BLA. This finding may provide a structural foundation to study the neural circuit mechanisms underlying sex differences in motivational and emotional behaviors and related psychiatric dysfunctions.

## Methods

### Animals

Male and female heterozygous TH-Cre mice [53] (70–105 days of age) in the C57BL/6 J strain were acquired from Charles Gerfen (NIMH, Bethesda, MD) and housed in a climate-controlled vivarium. All animals used in this study were housed in filter-top cages on ventilated racks with both males and females on each rack in the same room. Subjects were bred from heterozygous TH-Cre and wild-type (WT) parents. Mice were group housed under a 12/12-h light/dark cycle (lights on at 7 am) with ad libitum food and water. Experimental protocols were approved by the National Institute of Mental Health Animal Care and Use Committee and were in accordance with the National Institutes of Health Guidelines for the Care and Use of Laboratory Animals.

### Viral vectors

Two viral vectors, AAV9–CAG–FLEX–tdTomato (Plasmid #28306, Addgene) and AAV9–phSyn1(S)–FLEX–tdTomato–T2A–SypEGFP–WPRE (Plasmid #51509, Addgene), were generated by Boston Children’s Hospital Viral Core at titers of 1.0 × 10^13^ gc/mL and 4.3 × 10^13^ gc/mL, respectively. These vectors were injected into the VTA of TH-Cre mice to label dopaminergic axons (tdTomato) and highlight axonal synaptic boutons (SypEGFP). Because the fluorescent intensity of tdTomato expressed by AAV9–phSyn1(S)–FLEX–tdTomato–T2A–SypEGFP–WPRE was relatively weak for visualizing thin dopaminergic axons, AAV9–CAG–FLEX–tdTomato was co-injected to enhance tdTomato expression and facilitate axonal visualization.

### Stereotaxic surgery

Mice were anesthetized with 3% isoflurane, and 1–1.5% isoflurane was maintained throughout the surgery. Local anesthetic (0.05 mL xylocaine) was injected under the scalp prior to incision. We used fixed stereotaxic coordinates to target the VTA based on a mouse stereotaxic atlas [54]. The mouse head was fixed at 5° angle using a robotic stereotax (Neurostar), which provides good control of fixation angle. We manually measured four points on the skull (Bregma, Lamba, and two points placed half way between Bregma and Lamda and 2 mm to the left or right of the midline) and matched these surface points to a virtual atlas to adjust for skull angle. A cranial window was drilled 0.5 mm anterior and 0.5 mm lateral to Lambda (3.3 mm posterior to Bregma) on the surface of the skull [54]. Two viral vectors (1 µL FLEX–tdTomato–SynaptophysinGFP + 0.6 µL FLEX–tdTomato) were mixed prior to uptake in a glass injection needle, and 1 µL of the mixture was injected at a depth of 4.6 mm below the surface of the brain to target VTA in all the mice. Injection speed was set at a fixed rate (0.1 μL/min) and maintained by the Neurostar system. The needle was left for 5 min after the injection was complete before retraction. The incision was sealed with Vetbond (3 M) or surgical wound clip. Mice were placed on a heating pad until they began moving on their own, and then administered 1 mg/mL ketoprofen in sterile 0.9% saline through intraperitoneal (IP) injection (1 mL/ 25 g body weight). Ketoprofen was administered for 2 additional days post-surgery.

### Vaginal cytology

Vaginal cytology was assessed immediately before perfusion in female mice to avoid stress from repeated lavage [55]. Female mice were anesthetized using 3% isoflurane. A pipette was used to take up 25 µL 0.9% saline. The tip of the pipette was inserted 1–2 mm into the vaginal opening and the saline was pipetted in and aspirated back out 3–5 times. The liquid was then pipetted onto a 0.1% gelatin-subbed glass slide and smeared to prevent pooling in one spot. The liquid was allowed to dry, and then gently rinsed with deionized water to remove salt crystals. Slides were then visualized with a light microscope to determine the present cell types. For each mouse, the following cell types were assessed: nucleated epithelial cells, cornified squamous epithelial cells, or leukocytes. Proestrus was characterized by predominantly nucleated epithelial cells; estrus was characterized by clusters of cornified squamous epithelial cells; metestrus was characterized by predominantly leukocytes with some cornified squamous epithelial cells; diestrus was characterized by predominantly leukocytes, with some cornified squamous epithelial cells and some nucleated epithelial cells.

### Perfusion and immunohistochemistry

Mice were perfused with 0.9% saline, followed by 4% paraformaldehyde (PFA) in phosphate-buffered saline (PBS), and post-fixed overnight in 4% PFA at 4 °C. Perfusions were performed at roughly the same time of day (early afternoon ~ 1 pm) to limit variations in daily fluctuations of estradiol. Brains were sectioned on a vibratome into floating sections of 100 µm thickness. VTA sections were selected based on stereotaxic coordinates from − 3.1 bregma to − 3.6 bregma and BLA sections were selected from − 1.0 to − 3.0 bregma to cover the majority of VTA and BLA. Sections were permeabilized in 50% ethanol (EtOH) in PBS for 30 min then blocked for 1 h in 0.3% TritonX-100 + 5% Normal Donkey Serum (NDS). Sections were incubated in 5% NDS with 1:5000 rabbit–anti-TH (AB152, Millipore Sigma) overnight at 4 °C. Sections were washed 3 times with PBS before incubating in Alexa 488 Donkey–anti-Rabbit 1:200 (A-21206, ThermoFisher Scientific) for 1 h. Sections were washed 3 times with PBS and stored in PBS until mounting. After immunostaining, sections were mounted on 0.1% gelatin-subbed slides. Sections were allowed to dry completely, then rehydrated and cover-slipped with Aqua-Poly/Mount (Polysciences).

### Microscopy

Two microscopes were used to obtain images: an Olympus FV1000 confocal and a Zeiss 780 confocal. VTA images were taken with a 10× objective lens (Olympus, Zeiss) and BLA images were taken with both a 10× objective lens (Olympus) and a 20× objective lens zoomed to 25× magnification (Zeiss). An automated stage was used to tile and stitch the 25× images covering the BLA and surrounding area across 20 sections per mouse. Nine 25× BLA images were acquired per section, tiled 3 × 3, and Z-stacks were obtained with 10 images at 1 micron depth apart. Green and red channels were imaged sequentially to reduce the possibility of bleed through between channels.

### Image analysis

After image acquisition, all VTA and BLA images were arranged consecutively and matched between mice to ensure that the quantification region was comparable between mice. If a matching image was not found for all mice, that location was not used in any quantification. For Z-stacks, images were projected to a single plane by maximum intensity across the stack. ROIs for quantification were drawn in ImageJ around the labeled region and separated into red axon and green bouton channels for BLA and VTA images. Images were processed using a custom-written MATLAB (MathWorks) script with three subfunctions—one for detecting axons in the red channel of BLA images, one for detecting boutons in the green channel of BLA images, and one for detecting cells in both the red and green channels of VTA images. The axon detection function used Hessian filters to highlight line-like structures in the photomicrographs [56–58], and the filtered images were thresholded at 5 standard deviations (10× Olympus images) or 7 SD (25× Zeiss images) above image background in accordance with visual inspection. The detected line segments were then skeletonized to one pixel-width, so total pixel output would represent axon length. The bouton detection function used Laplacian-of-Gaussian filters to highlight point-like structures in the microscope [56, 59, 60], and the filtered images were thresholded at 5 SD (10× Olympus images) or 7 SD (25× Zeiss images) in accordance with visual inspection. The cell detection function used Laplacian-of-Gaussian filters to highlight round objects between 49 and 200 µm^2^ in size. The filtered images were thresholded at 3 SD in accordance with visual inspection, and the small structures below the size limit were removed.

### Statistical analysis

Labeling intensity differences between multiple brain regions were compared using one-way repeated measures ANOVA with multiple comparisons corrected by controlling the false discovery rate (0.05) in Prism (GraphPad). The effect size for repeated measures ANOVA was reported using eta squared. Both axon and bouton density in the BLA were normalized to the number of labeled cells at the VTA injection site. Sample size for viral labeling comparison is based on previous anatomical analysis and preliminary findings in bouton density [56]. Statistical differences between males and females were determined with a two-tailed Student’s *t* test in Prism (GraphPad) when normality and equality of variance were achieved. When variances were not equal, unpaired *t* tests with Welch’s correction were used. A *p* value below 0.05 was considered statistically significant. Effect sizes are reported using Cohen’s d.

## Results

### Viral labeling of dopaminergic projections from VTA to forebrain target regions

**Fig. 1:**
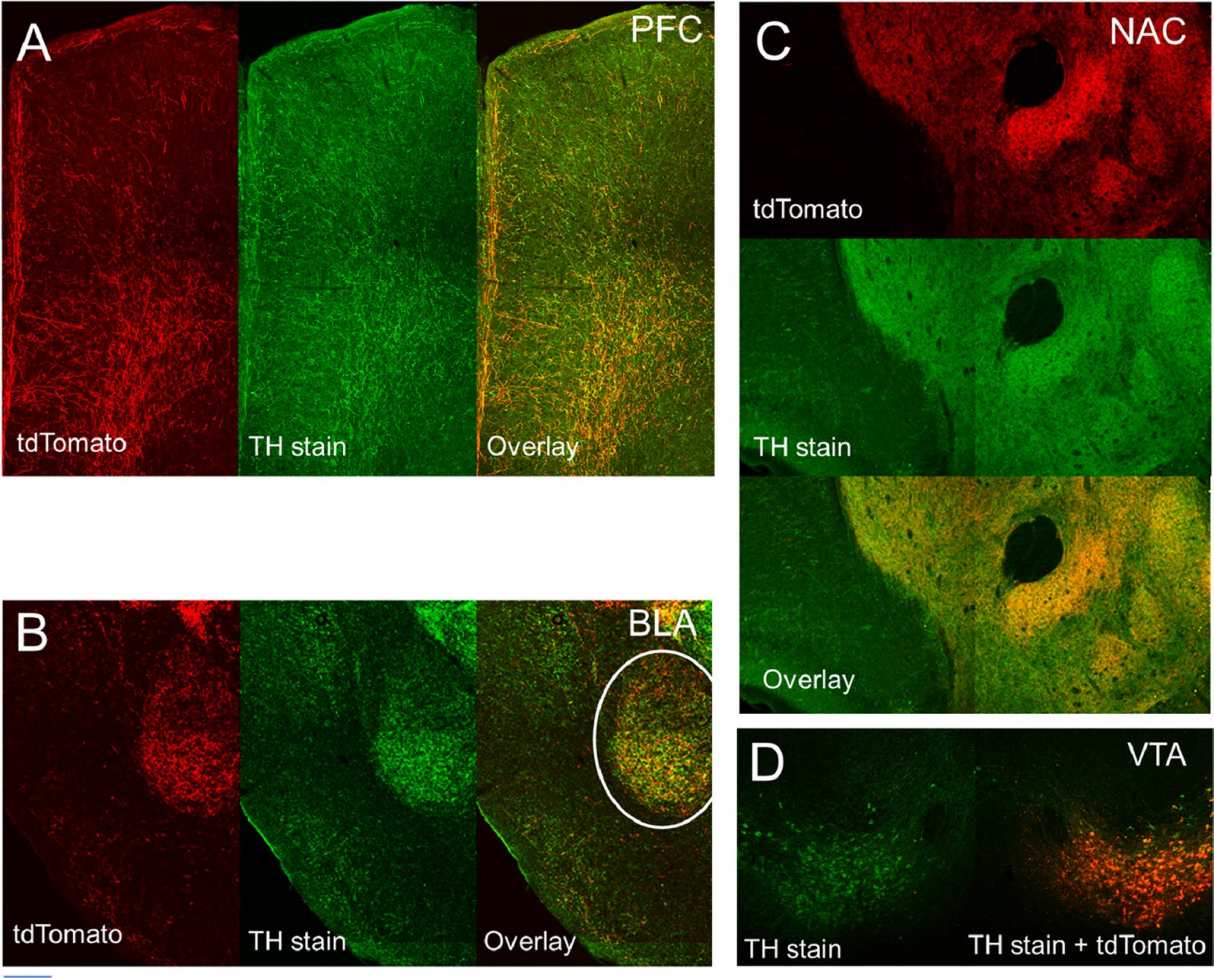
Viral labeling of dopaminergic projections from VTA to forebrain target regions. Tyrosine hydroxylase (TH) immunostaining on brain sections from TH-Cre mice with AAV–CAG–Flex–tdTomato viral injection into the ventral tegmental area (VTA) shows extensive co-labeling between TH (green) and tdTomato (red). Confocal fluorescent microscopy was used to image several major forebrain projection targets of VTA dopamine neurons. **A** Prefrontal cortex (PFC) [2.0 bregma]. **B** Basolateral amygdala (BLA) [− 2.0 bregma]. **C** Nucleus accumbens (NAC) [1.1 bregma]. **D** VTA images at the injection site of tdTomato virus (red) [− 3.1 bregma]. Images were acquired at 10 × magnification. The scale bar represents 500 µm

To probe the potential anatomical sex differences in specific dopaminergic circuits, we used a combination of cell-type specific transgenic driver mice and stereotaxically injected viral reporters. Specifically, a transgenic mouse line expressing Cre recombinase under the control of Tyrosine Hydroxylase (TH) promoter was used to restrict Cre-dependent reporter expression to cells that express TH, an enzyme required for dopamine synthesis [53, 56]. While this transgenic method alone would not exclude norepinephrine (NE) cells that also express TH, stereotaxic injection of a Cre-dependent viral reporter (AAV9–CAG–FLEX–tdTomato) into the VTA, which does not contain NE cells, limited reporter labeling to dopaminergic cells in the VTA. Our previous study using this viral labeling method has demonstrated high efficiency and specificity of this approach to label dopaminergic cells in VTA [56]. To further examine the extent of viral reporter labeling of dopaminergic projections to forebrain target regions, including BLA, NAC and PFC [34, 52], we performed TH immunostaining on brain sections containing these regions after VTA viral injection [56, 61]. Using confocal fluorescent microscopy on coronal brain sections, we found that the viral reporter co-labeled extensively with the TH-positive axons in PFC, BLA, and NAC (Fig. 1).

### BLA shows an intermediate level of dopaminergic axon labeling compared to NAC and PFC

**Fig. 2:**
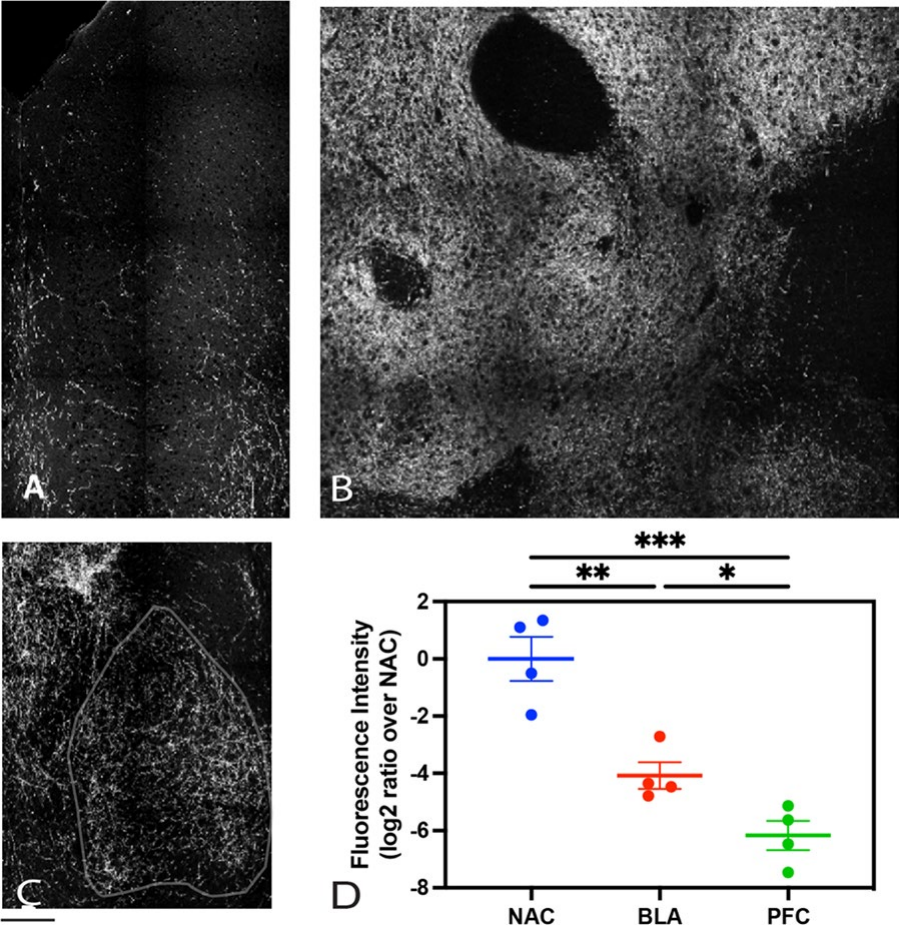
BLA shows an intermediate level of dopaminergic axon labeling compared to NAC and PFC. **A**–**C** Confocal images showing tdTomato labeled dopaminergic axons in **A** prefrontal cortex (PFC), **B** nucleus accumbens (NAC), and **C** basolateral amygdala (BLA). The contour of BLA was drawn with a grey line according to Allen Mouse Brain Atlas. **D** Quantification of tdTomato fluorescence intensity in each target region. Individual intensity values were normalized to average NAC intensity and expressed as log2 ratios. There is a significant difference across regions: one-way repeated measures ANOVA, F(2,6) = 19.96, *p* = 0.0022, effect size (eta squared) = 0.8693, *n* = 4 mice per region. Multiple comparison tests indicate: **, NAC vs. BLA, *p* = 0.0033, effect size = 3.21; *, BLA vs. PFC, *p* = 0.0279, effect size = 2.15; ***, NAC vs. PFC, *p* = 0.0008, effect size = 4.73. Scale bar represents 500 µm. Error bars represent Mean ± SEM

Visual inspection of tdTomato-labeled dopaminergic projections in forebrain regions suggested highest labeling in NAC, intermediate labeling in BLA, and sparse labeling in PFC (Fig. 2A–C). Because NAC labeling density was too high to allow segmentation of single axons in optical images, we measured average tdTomato fluorescence intensity per pixel in each brain region-of-interest (ROI). In addition, the fluorescent intensity in each forebrain target was normalized to VTA labeling to control for variations in viral expression within each brain. The resulting values were then divided by average NAC intensity and expressed as log2 ratios for inter-regional comparisons. Quantitative analysis of these ratios revealed a significant difference across NAC, BLA and PFC regions (one-way repeated measures ANOVA, F(2,6) = 19.96, *p* = 0.0022, effect size (eta-squared) = 0.8693). NAC labeling was significantly higher than BLA, and BLA labeling was significantly higher than PFC (multiple comparison tests, NAC vs. BLA, *p* = 0.0033, effect size = 3.21; BLA vs. PFC, *p* = 0.0279, effect size = 2.15; NAC vs. PFC, *p* = 0.0008, effect size = 4.73). The intermediate level of BLA labeling provides a useful balance between the quantification requirements for single-axon morphology analysis, which needs sparser labeling, and efficient axon sampling, which needs denser labeling. Because dopaminergic projections to BLA have received little in-depth anatomical characterization in previous research, we focused the rest of this study on the BLA.

### tdTomato and SypGFP labels distinguish between dopaminergic axons and boutons

**Fig. 3:**
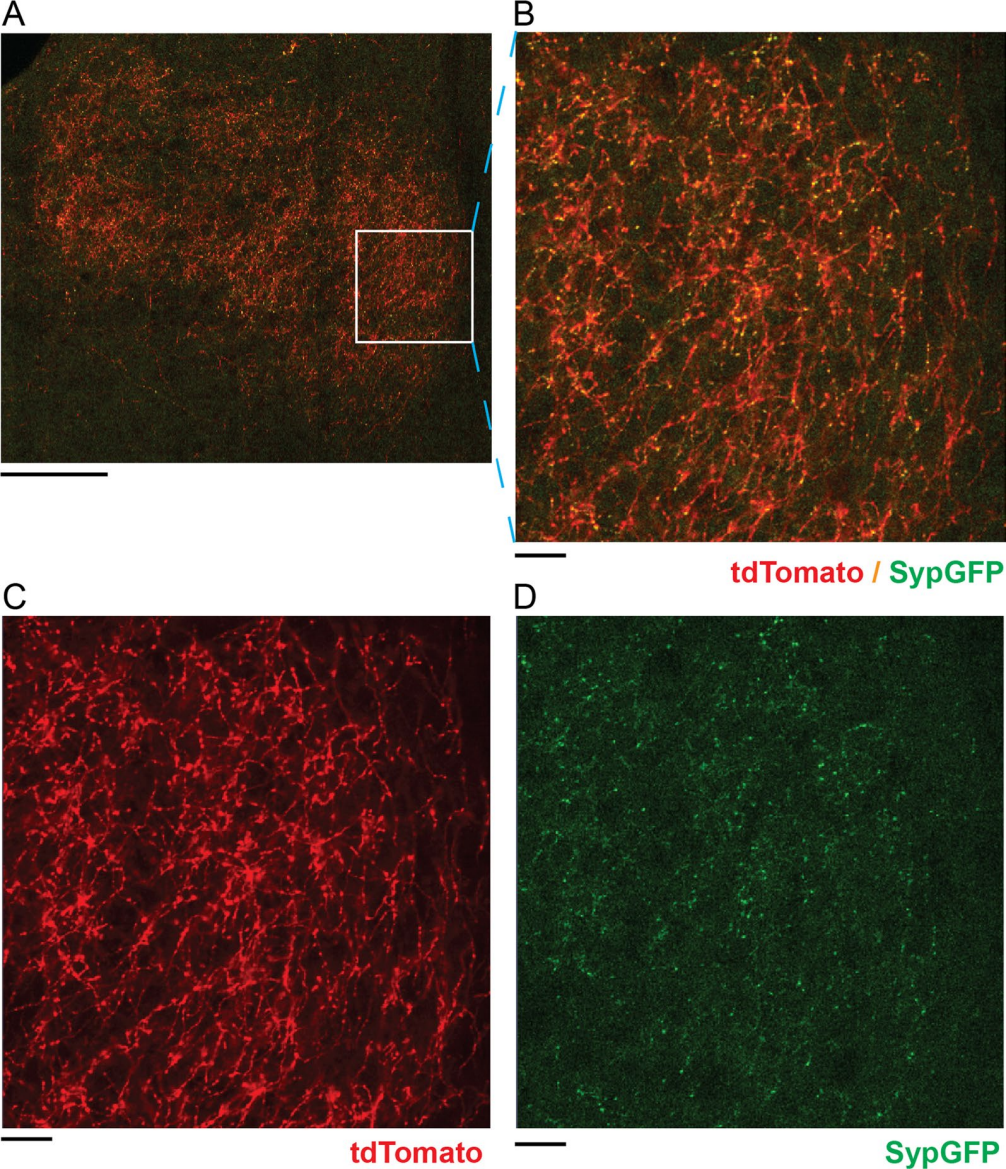
tdTomato and SypGFP labels distinguish between dopaminergic axons and boutons. **A** Representative confocal image of the entire BLA area in a coronal section at − 2.06 bregma showing dopaminergic axons and boutons labeled by tdTomato (red) and SypGFP (green). **B**–**D** Area inside the white box in **A** is further enlarged for display in both red and green channels (**B**), red channel only (**C**), and green channel only (**D**). The image was acquired at 25× magnification. Scale bars represents 200 µm in (**A**) and 25 µm in **B**, **C**

Dopaminergic axons contain local structural enlargements called boutons, where dopamine is typically released in synaptic vesicles [62, 63]. While both boutons and axons can be labeled with tdTomato alone, it is difficult to set an appropriate intensity threshold to separate axons and boutons labeled in the same color for automated image analysis. Previous studies have shown that a reporter created by tagging the synaptic vesicle protein, Synaptophysin, with green fluorescent protein (SypGFP) can serve as a marker for presynaptic terminals [64, 65]. To test this reporter for labeling dopaminergic boutons, we co-injected Cre-dependent tdTomato and SypGFP AAV vectors into the VTA of TH-Cre mice. Confocal images of BLA showed that dopaminergic axons and boutons are clearly labeled by tdTomato and SypGFP (Fig. 3A, B). While tdTomato filled up linear axonal projections (Fig. 3C), SypGFP concentrated in boutons in a punctate pattern (Fig. 3D). Use of two different reporters to label axons and boutons separately allows structural quantification to be performed in parallel channels and improves bouton detection.

### Exploration of the sex difference in VTA-to-BLA dopaminergic projections

**Fig. 4:**
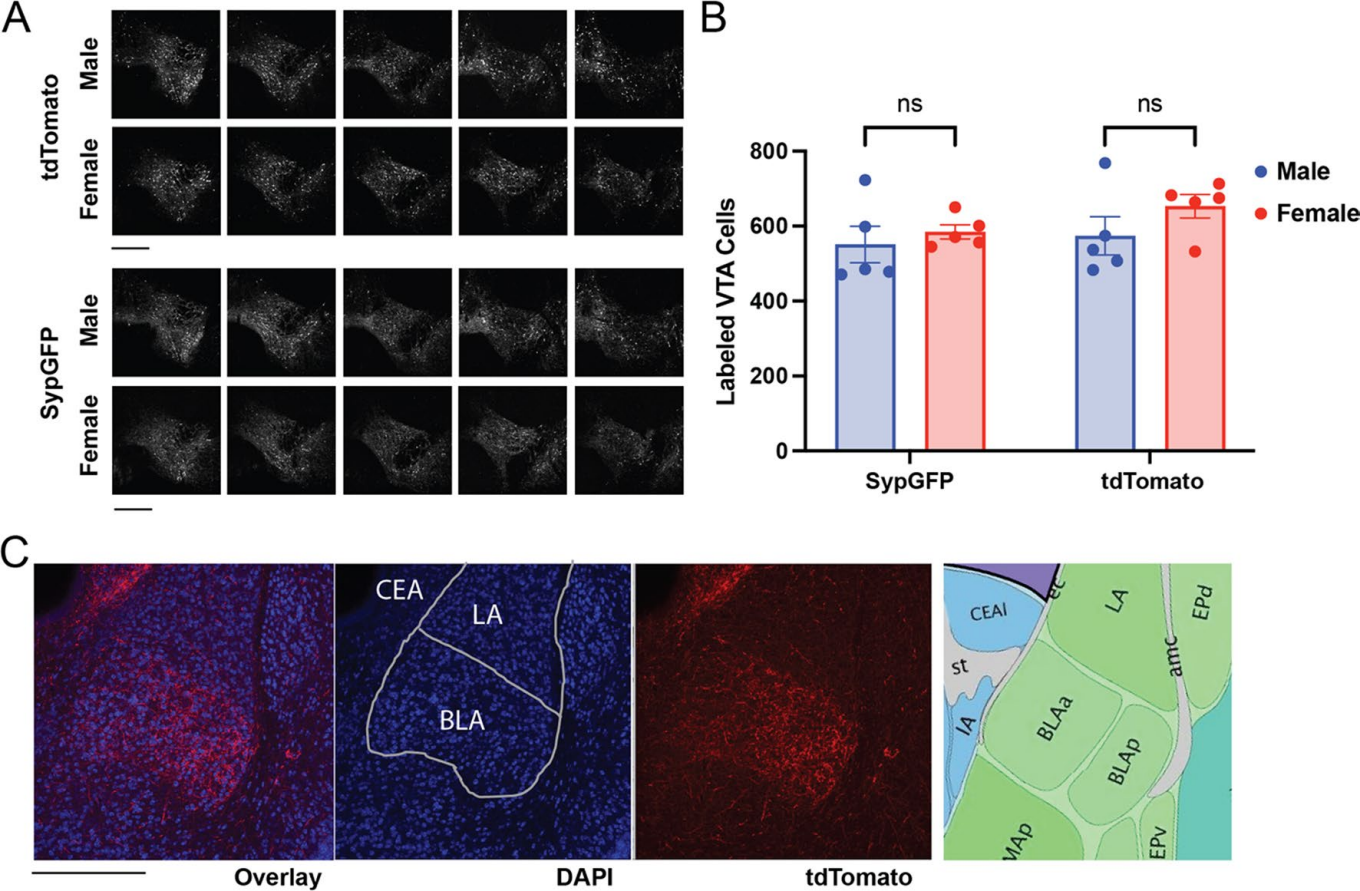
Characterization of the extent of viral labeling in VTA and BLA. **A** Representative confocal images of coronal serial sections of VTA from male and female TH-Cre mice labeled with Cre-dependent tdTomato or SypGFP virus. **B** Quantification of viral-labeled cells in VTA showed no sex difference in SypGFP or tdTomato expression. Two-way ANOVA, Sex F(1,16) = 1.321, *p* = 0.2673; Label F(1,16) = 2.020, *p* = 0.1745; Interaction F(1,16) = 0.3318, *p* = 0.5726. Post hoc comparison, Male vs. Female, SypGFP *p* = 0.8051, tdTomato *p* = 0.3228. **C** Representative confocal images of coronal sections of BLA labeled with tdTomato (red) and nuclei counterstain DAPI (blue). BLA boundaries were defined in DAPI-counterstained sections. Viral labeling from VTA projections closely matched the cytoarchitectural boundary of BLA (including both the anterior (BLAa) and posterior (BLAp) parts of the basolateral amygdala in Allen Mouse Brain Atlas, right panel). Scale bars in **A**, **C**: 500 µm

To explore to what extent sex differences in dopaminergic projections from VTA to BLA exist in adult mice, we co-injected Cre-dependent tdTomato and SypGFP AAV vectors into the VTA of both male and female TH-Cre mice (Fig. 4A). Quantification of viral-labeled cells in the VTA showed no sex difference in SypGFP or tdTomato labeling (Fig. 4B, two-way ANOVA, Sex F(1,16) = 1.321, *p* = 0.2673; Label F(1,16) = 2.020, *p* = 0.1745; Post hoc comparison, Male vs. Female, SypGFP *p* = 0.8051, tdTomato *p* = 0.3228). The comparable VTA cell labeling across sex groups indicates consistent viral coverage.

In the amygdala, viral labeling from VTA projections closely matched the cytoarchitectural boundary of BLA, which was defined in DAPI-counterstained sections (Fig. 4C). Dopaminergic projections concentrated in BLA with a distinct border from the adjacent lateral amygdala (LA) and basomedial amygdala (BMA). Dense dopaminergic labeling was also present in central amygdala (CEA). However, the labeling density in CEA is too high for axonal segmentation, which is similar to the situation in NAC and may be related to the striatal origin of CEA [41].

**Fig. 5:**
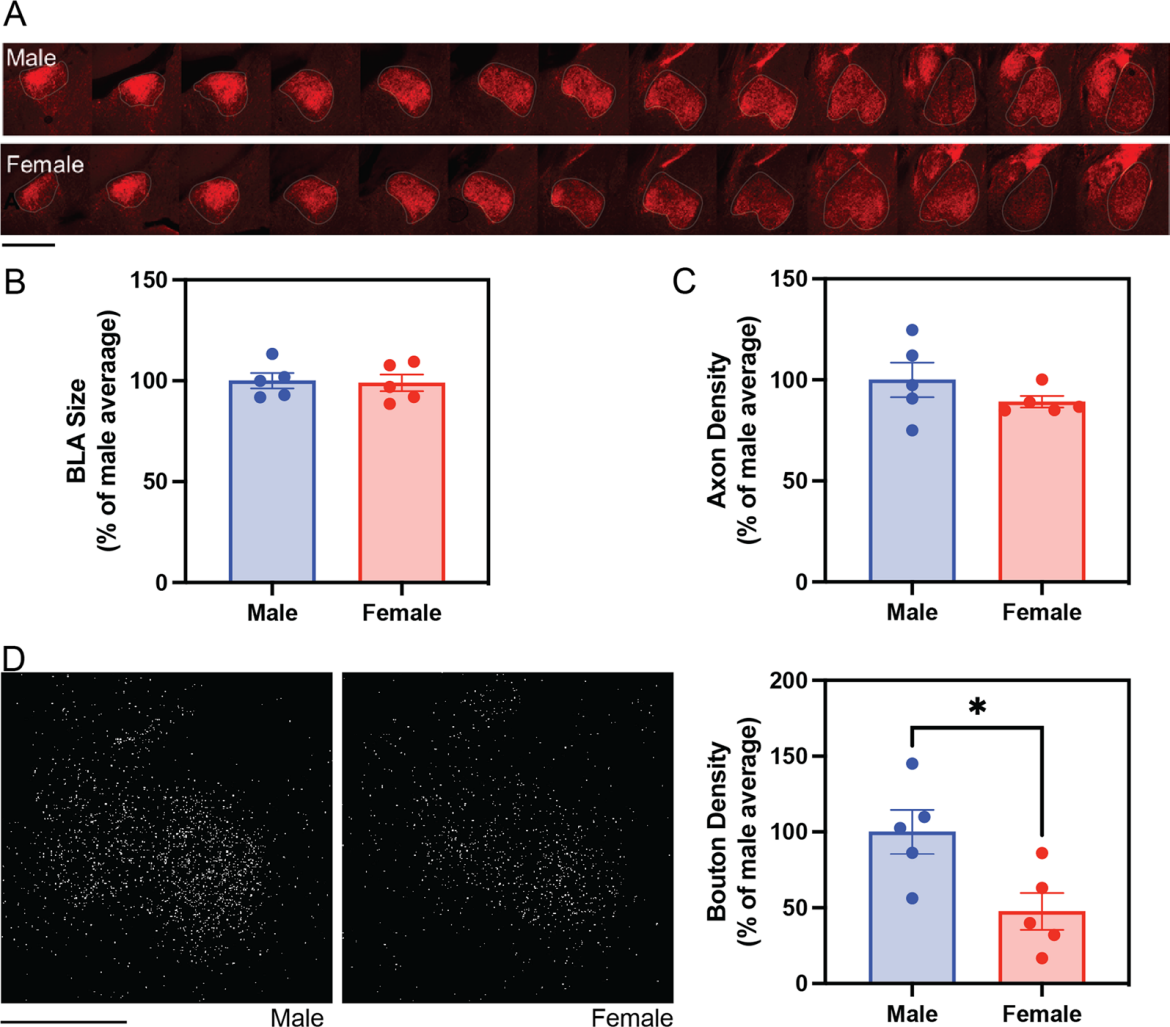
Exploration of sex difference in VTA-to-BLA dopaminergic projections reveals increased bouton density in males. **A** Representative confocal images of coronal serial sections of BLA from male and female mice. VTA-to-BLA dopaminergic projections were labeled by tdTomato in red. The contours of BLA were drawn with grey lines according to the DAPI-stained cytoarchitecture and Allen Mouse Brain Atlas. **B** No sex difference in BLA size was observed (t (8) = 0.19, *p* = 0.85). **C** Dopamine axon density in BLA shows no sex difference (t (8) = 1.2, *p* = 0.26). **D** Representative binarized images of detected dopaminergic boutons (SypGFP +) projected from anterior-posteriorly matched serial sections of BLA in male and female mice. Male mice have significantly higher densities of dopaminergic boutons in BLA than female mice (t (8) = 2.76, *p = 0.0246, effect size = 1.75). The estrous stage in females was estimated approximately by vaginal cytology. All five female mice in this cohort were coincidentally in estrus at the time of sample collection. Images were acquired at 10× magnification. Scale bars represent 800 microns in **A** and 500 µm in **D**. Bars represent Mean ± SEM

To account for potential sex differences in the volume of BLA, regions containing BLA were imaged across a longitudinal series of coronal sections (Fig. 5A). The size of BLA was approximated by summing the cross-sectional area from the same range of serial sections for each mouse, and these size values were expressed as percentages of the average value of male BLA size for cross-sex comparison. No sex difference was detected in the size of BLA (t (8) = 0.19, *p* = 0.8533; Fig. 5B).

To assess the potential sex differences in dopaminergic axon and bouton density in BLA, we used custom-written MATLAB scripts (see details in Methods) to detect and quantify labeled axons and boutons. Axons were detected by drawing ROIs around the BLA in the red channel and isolating continuous line segments using Hessian line filters [56, 61]. Detected axons were skeletonized to a width of one pixel to focus on axon length quantification. Boutons were detected in BLA by applying a Laplacian-of-Gaussian filter to the green channel to isolate bright point-like objects of 1 µm in size. In addition, reporter-labeled dopaminergic cells were counted at the VTA injection site to determine viral labeling efficiency. Axon density was reported as the total number of axon pixels summed across BLA sections and normalized to the number of tdTomato labeled cells in the VTA of each mouse. Bouton density was reported as the total number of boutons summed across BLA sections and normalized to the number of sypGFP labeled cells in the VTA of each mouse. These density values were then expressed as percentages of the average value of male axon or bouton density for cross-sex comparison. Using percent difference between sex groups, rather than raw image pixel units, facilitates data interpretation across different batches of experiments.

We compared dopaminergic axon density in BLA between adult male and female mice but observed no sex difference (t (8) = 1.2, *p* = 0.264, Fig. 5C). However, the density of boutons in the BLA was significantly higher in males than in females (t (8) = 2.8, *p* = 0.0246, effect size = 1.75, Fig. 5D). On average, bouton density in females is approximately 50% of that observed in males. In addition, to explore if dopaminergic bouton density may change with hormonal cycles in females, we assessed estrous stages from vaginal cytology samples collected prior to collection of brain tissue; however, all five female mice in this cohort were coincidentally in estrus at the time of sample collection, thus no estrous cycle effect could be assessed. Our findings from this cohort of mice suggest that while dopaminergic axon density does not differ between males and females in the BLA, dopaminergic bouton density is higher in male mice compared to female mice.

### Confirmation of increased BLA dopaminergic bouton density in males compared to females

**Fig. 6:**
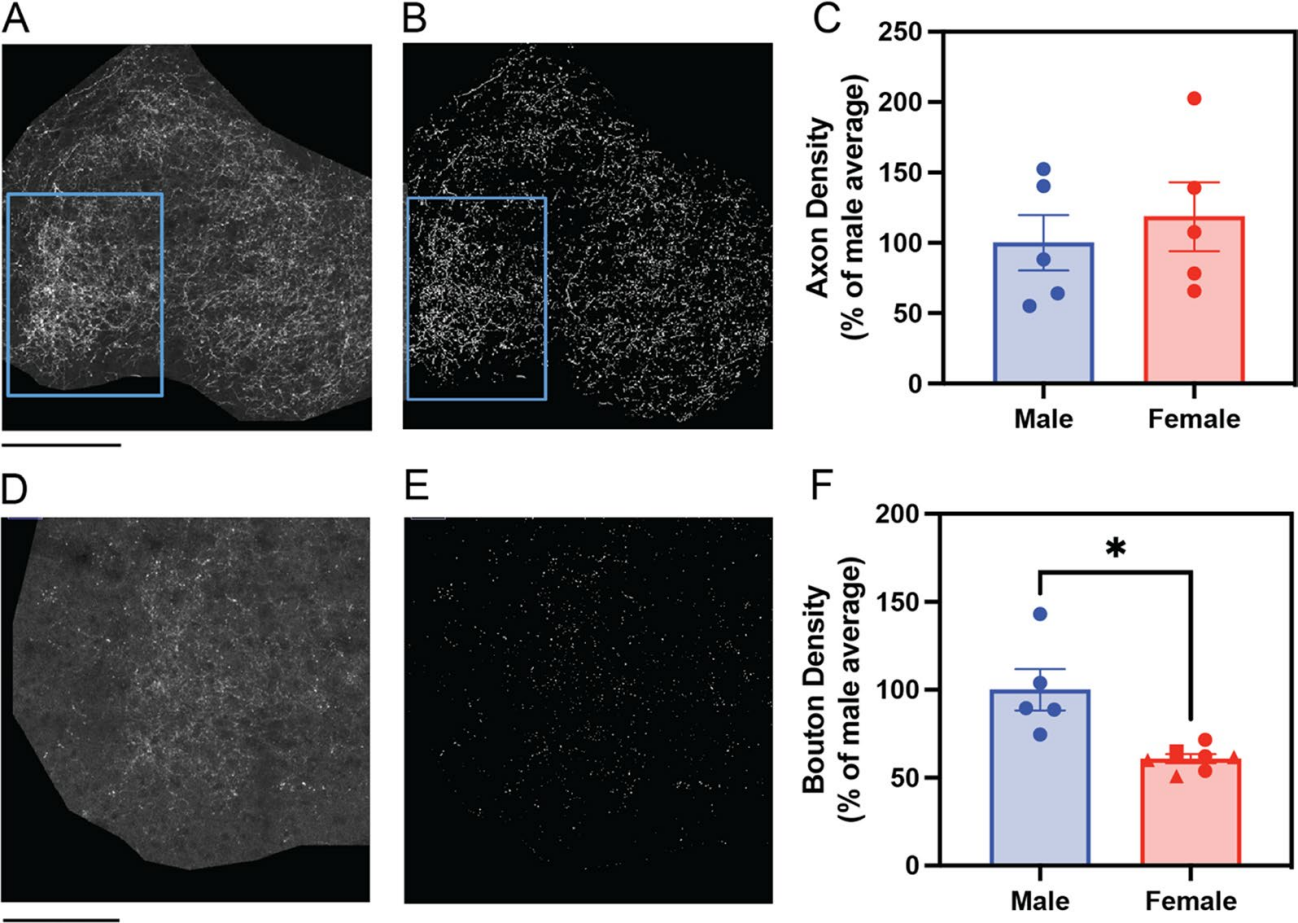
Confirmation of increased BLA dopaminergic bouton density, but not axon density, in males compared to females. **A** Representative confocal image of tdTomato-labeled dopaminergic axons (white) in a single section of basolateral amygdala (BLA) from the second cohort of mice. The contour of BLA was drawn according to the DAPI-stained cytoarchitecture and Allen Mouse Brain Atlas. The pixels outside the BLA contour were set to zero intensity (dark) for automated quantification. The blue box indicates the area that will be enlarged for display of labeled boutons in **D**. **B** Binarized image of detected axons in **A**. **C** No sex difference in dopaminergic axon density was detected (t (8) = 0.59, *p* = 0.57, *n* = 5 mice per sex). **D** Representative confocal image of SypGFP-labeled dopaminergic boutons (white) in a single section of basolateral amygdala (BLA) from the second cohort of mice. This region is enlarged from the blue-boxed area in A for display. **E** Binarized image of detected boutons in **D**. **F** Male mice have significantly higher densities of dopaminergic boutons in BLA than females (t (4.4) = 3.26, *p = 0.027, with Welch’s correction for unequal variance, effect size = 2.05, *n* = 5 male mice and *n* = 7 female mice). The estrous stage in females was estimated approximately by vaginal cytology and indicated in the plot by the shape of markers: estrus (circles, *n* = 3), diestrus (triangles, *n* = 3) and proestrus (square, *n* = 1). Axon and bouton density were calculated across 20 matched BLA coronal sections (− 3.0 bregma to − 1.0 bregma) from each mouse and expressed as percentage of the average axon or bouton density in males. Images were acquired at 25× magnification. Scale bars represent 500 microns (**A**, **B**) and 250 microns (**D**, **E**). Bars represent Mean ± SEM

To confirm the observed sex differences of dopaminergic bouton density in BLA, a second cohort of brain samples was collected from adult male and female TH-Cre mice that received co-injection of Cre-dependent tdTomato and SypGFP AAV vectors in VTA. In addition, the confocal imaging magnification was increased by 2.5-fold to enable more sensitive detection of axons (Fig. 6A, B) and boutons (Fig. 6D, E). Consistent with the findings from the previous cohort, BLA showed no sex difference in dopaminergic axon density in the second cohort (t (8) = 0.59, *p* = 0.571; Fig. 6C), but males showed significantly higher bouton density than females (t (4.4) = 3.26, *p* = 0.027, with Welch’s correction for unequal variance, effect size = 2.05, Fig. 6F). For axon comparison, male and female groups were equal in size (*n* = 5 mice/sex). For bouton comparison, an additional 2 female mice were added to increase representations in different estrous stages. The estrous stage in females was estimated approximately by vaginal cytology prior to collection of brain tissue to avoid the stress of repeated lavage. BLA bouton density appeared to be similar between the estrus (*n* = 3), proestrus (*n* = 1) and diestrus (*n* = 3) groups and lower than that in males (Fig. 6F, data points corresponding to different estrous stages are coded by distinct marker shapes). No metestrus females were available in this cohort. These results confirmed the increased dopaminergic bouton density in male BLA compared to female BLA, but future studies involving repeated measures of estrous cycles in more females are needed to assess the potential effect of estrous stage on bouton density.

## Discussion

The focus of this study was to determine if male and female mice show anatomical differences in dopaminergic projections from VTA to BLA. Here, we have identified a previously unknown subcellular sex difference in this circuit—namely, a greater density of synaptic boutons on VTA-to-BLA dopaminergic projections in male mice compared to female mice. We also report dopaminergic axons are concentrated in BLA compared to adjacent LA and BMA areas, but BLA axon densities do not differ between male and female mice. Thus, the increased density of dopaminergic boutons in the BLA of male mice may provide a concrete anatomical substrate underlying the higher extracellular dopamine level previously reported in the BLA of male rats compared to female rats [27].

Anatomical differences in the density of dopaminergic synaptic boutons may result in a different level of dopamine release in males and females if other physiological parameters, such as the firing rate of dopamine neurons, are comparable. These possibilities could be investigated in future studies of both sexes by electrophysiological recording of dopamine neuron firing activities in VTA, and measurement of dopamine release via microdialysis or fluorescent dopamine sensors in BLA [66–68]. Particularly, combining optogenetic stimulation of VTA-to-BLA projections with fiber photometry of dopamine release in BLA would provide a precise assessment of the physiological correlates of this anatomical sex difference identified in this study.

It will also be important to determine if the anatomical sex difference in BLA translates to a behavioral sex difference. Earlier rodent behavior studies have revealed sex differences in several tasks that may be dependent on BLA function, such as classical fear conditioning, active avoidance, auditory fear discrimination, fear generalization, and fear extinction [69–73]. Pharmacological modulation of dopaminergic signaling suggested that the amygdala dopaminergic system is involved in the formation and expression of fear conditioning [74]. Furthermore, several recent studies using projection-specific optogenetic, chemogenetic, and optical imaging techniques reported that VTA-to-BLA dopaminergic projections provide gating of salient visual or somatosensory cues and regulate anxiety-like behaviors [35, 49, 50]. However, sex differences in VTA-to-BLA circuit function were not examined in these studies. Intriguingly, a recent study reported a sex difference in FOS gene expression in the basal amygdala during fear conditioning [75]. It will be interesting to investigate in future studies whether the sex difference in dopaminergic synaptic boutons may affect dopamine release in BLA under these behavioral conditions and contribute to physiological and behavioral sex differences.

We report an anatomical sex difference in adult mice, but when this difference emerges during development is unknown. Sex differences in the brain may appear as a result of prenatal organization by gonadal steroid hormones, or emerge during postnatal maturation [15, 76]. Early emerging sex differences typically include rate of neurogenesis and dendritic growth patterns, while differences emerging later in development often appear in the activational states of specific circuits or cellular subpopulations [77–79]. Dopaminergic axons from the midbrain reach the forebrain targets during prenatal development, but the innervation density shows a protracted increase during postnatal development and can be modulated by neural activity and behavioral experiences [56, 80–82]. There is also a substantial body of literature showing the effects of androgen removal on prefrontal dopaminergic circuits in adult male rats, as well as the role for estrogen in the sex differences in hypothalamic dopamine neurons and sexual behavior of male mice [29, 83–88]. The role of both androgens and estrogens in the maintenance of anatomical sex difference of BLA dopaminergic innervations awaits to be explored in future studies. Additional avenues for future research include investigating when the anatomical sex difference in VTA-to-BLA projections emerges during development, and to what extent developmental circulating hormones may shape this difference.

## Perspectives and significance

Our results lend support to the general hypothesis that the forebrain dopaminergic system may differ between males and females and contribute to sex differences in motivated behaviors [89]. Our findings raise specific questions about whether males and females show a difference in midbrain regulation of BLA function, when such a sex difference emerges during development, and which molecular factors underlie the differences between males and females. Answering these questions will improve our understanding of the roles the dopaminergic circuit plays in psychiatric risk, as well as provide possible targets for differential therapeutic intervention in men and women.

## Conclusions

Our study has identified an increased density of dopaminergic synaptic boutons in VTA-to-BLA projections in male mice compared to female mice. This anatomical sex difference is specific to dopaminergic bouton density, but not to dopaminergic axon density or the size of the BLA. These findings may provide an anatomical foundation to study the neural circuit mechanisms underlying sex differences in motivational and emotional behaviors and related psychiatric dysfunctions.

## Data Availability

The data that support the findings of this study are available from the corresponding author upon reasonable request.
